# Attentional Processing Biases in Young People With Binging and Purging Behavior

**DOI:** 10.1002/brb3.70322

**Published:** 2025-02-16

**Authors:** Aglaia Freccero, Victoria Burmester, Rachel Rodrigues, Alessia Gallucci, Dasha Nicholls, Martina Di Simplicio

**Affiliations:** ^1^ Division of Psychiatry, Department of Brain Sciences Imperial College London London UK; ^2^ IRCCS Fondazione Don Carlo Gnocchi Milan Italy

**Keywords:** adolescents, attentional bias, binging, dot‐probe task, eating disorder, purging, young people

## Abstract

**Objective:**

Binging and purging are transdiagnostic features of eating disorders (EDs). Attentional biases (ABs) toward food and body shape cues and negative affect (NA) are associated with ED psychopathology. These ABs might also be present in people with subthreshold ED not meeting full diagnostic criteria. We investigated ABs to food and body shape cues and the interaction between ABs and NA in young people with binge/purge behavior (B/P group) and healthy controls (HC group). Our B/P sample consisted of individuals with threshold and subthreshold ED, including participants with BN, AN‐R, and AN‐B/P.

**Method:**

We conducted two studies. Study 1 recruited *n* = 54 HC and *n* = 53 B/P participants aged 16–25, and Study 2 recruited *n* = 73 HC and *n* = 72 B/P participants. In Study 1, ABs toward food and body shape cues were compared between B/P versus HC participants using a pictorial dot‐probe task. In Study 2, ABs were compared between B/P versus HC participants after NA induction using the Cyberball social exclusion task. Indexes of attentional engagement and disengagement were computed.

**Results:**

There was a main effect of cue type on attentional engagement at 0.2 s (*p* = 0.006, $\eta_{p}^{2}$ = 0.075) and 2 s (*p* = 0.040, $\eta_{p}^{2}$ = 0.043), and attentional disengagement at 2 s (*p* = 0.006, $\eta_{p}^{2}$ = 0.077) in Study 1. Findings were not replicated following NA induction in Study 2. No main effect of group or group × cue type interaction was found.

**Discussion:**

Our results disagree with previous research supporting the importance of ABs toward food and body shape cues in young people with threshold and subthreshold EDs and suggest these might not constitute a relevant target in the treatment of ED behavior. However, due to a heterogeneous approach to measuring ABs and multiple types of AB being described in EDs, further research is needed to clarify whether ABs map onto transdiagnostic models of behavioral dysregulation.

## 1 Introduction

Binging and purging are transdiagnostic disordered eating behaviors that may be present in binge‐eating disorder (BED), bulimia nervosa (BN), anorexia nervosa (AN), and other specified feeding and eating disorders (OSFED). EDs are frequent among young people (Silén and Keski‐Rahkonen 2022). In 2023, 12.3% of 11–16‐year‐olds and 59.4% of 17–19‐year‐olds in the United Kingdom had eating problems (NHS Digital 2023), and up to 40% of 16‐year‐old girls show disordered eating behavior (Bould et al. 2018). Shared psychopathology across ED diagnoses includes interpersonal difficulties, emotional dysregulation, and cognitive biases (Fairburn et al. 2003). EDs often co‐occur with other psychiatric diagnoses (Hambleton et al. 2022) and dysregulated behaviors such as self‐harm (Warne et al. 2021) and substance misuse (Bahji et al. 2019). The adoption of a transdiagnostic model to explore cognitive processes underlying dysregulated behaviors could improve treatment outcomes and inform the development of new interventions (Lynch et al. 2021).

Data from neuroimaging, neurocognitive, genetic, and translational animal studies have shown the presence of specific biases in reward processing and attention in individuals with EDs (Fusar‐Poli et al. 2019), similar to those observed in individuals with substance use disorder (Lozano‐Madrid et al. 2020). Sensitization‐related neuroadaptations explaining altered reward systems and craving in substance abuse could also explain motivational processes underlying ED and contribute to the onset and maintenance of binging and purging behavior (Berridge 2009; Berridge et al. 2010). According to the incentive sensitization theory of addiction, salient cues become conditioned and predictive of reward (Robinson and Berridge 1993; Berridge and Robinson 2016), resulting in preferential allocation of attentional resources toward them, so‐called attentional bias (AB) (MacLeod et al. 1986).

AB is a multidimensional construct and can be distinguished into different measurable components: attentional engagement (AE), disengagement (AD), and avoidance (Koster et al. 2004, 2006). In individuals with EDs, attention might be differentially attended to food and body shape cues and contribute to the overvaluation of food and preoccupation with body weight and shape (Stojek et al. 2018; Ralph‐Nearman et al. 2019; Stott et al. 2021). Evidence suggests that attentional patterns in EDs vary based on stimulus characteristics (low‐ vs. high‐calorie foods; higher vs. lower body mass index) (Stott et al. 2021). ABs also might differ among ED subtypes. In BN, research shows poorer disengagement from low‐BMI depictions of others and intentional avoidance of high‐BMI depictions (Blechert et al. 2009), while individuals with AN tend to exhibit conscious attentional avoidance toward food‐related stimuli (Giel et al. 2011; Sfärlea et al. 2023; Puttevils et al. 2023). Patients with AN‐R and AN‐B/P, respectively, show vigilance and avoidance in their attention allocation to eating disorder‐related and anxiety‐related threat words (Mann et al. 2018).

However, overall, previous literature shows inconsistent results in AB patterns in ED, possibly due to the variety of AB conceptualization, experimental paradigms used, sample characteristics, and modifying factors considered. It is, therefore, unclear whether ABs are a viable treatment target, and the efficacy of interventions targeted at modifying eating behavior, such as attention bias modification therapy, remains marginal (MacLeod et al. 2002; Renwick et al. 2013; Engel et al. 2019; Hardman et al. 2013). Moreover, there is a lack of research investigating the role of attentional processes in disordered eating in young people with threshold and subthreshold ED, so further investigation is required to understand how ABs map onto transdiagnostic models of behavioral dysregulation.

Attentional processes and affect regulation could act interdependently in the maintenance of repetitive dysregulated eating behaviors. The experience of negative emotions (negative affect; NA) increases leading up to a binging/purging episode, while their intensity is briefly reduced after an episode (Leehr et al. 2015). Affective changes activate cognitive expectations of improvements in emotional states following binging/purging and impact reward‐related cognition, thus serving to maintain this behavior (Smith et al. 2020). The interplay between interpersonal dysfunction and eating patterns has also been described as a possible source of indirect NA involved in loss of control of overeating (Ansell et al. 2012; Rieger et al. 2017). The role of NA may also be a shared feature contributing to dysregulated behaviors across diagnoses.

Thus, as part of a wider study investigating motivational biases in dysregulated behavior (Rodrigues et al. 2023), we aimed to characterize ABs in transdiagnostic dysregulated eating behaviors. In Study 1, we examined whether ABs toward food and body shape cues are present in young people (aged 16–25) who binge/purge (B/P group) compared to healthy controls (HC group) using a pictorial dot‐probe task. In Study 2, we examined the interaction between NA, induced by exposure to a social exclusion paradigm, and ABs to food and body shape cues in the same population.

We hypothesized that in Study 1, participants in the B/P group would experience increased engagement and maintenance of attention toward food and body shape cues compared to the HC group. We predicted that in Study 2, B/P participants would experience greater aversive emotions than the HC group following the social exclusion paradigm and that this state would translate into increased engagement and maintenance of attention toward food and body shape cues compared to the HC group. We also explored whether the strength of ABs was associated with ED symptomatology in both studies.

## 2 Methods

### 2.1 Participants

Participants were young people (16–25 years old) engaging in binging and/or purging behavior (B/P group) and the HC group. A total of *N* = 107 participants (*n* = 54 HC and *n* = 53 B/P) and *N* = 145 (*n* = 73 HC and *n* = 72 B/P) were recruited from across the UK community, respectively, for Studies 1 and 2. The studies were advertised in public spaces using posters and on social media platforms to direct participants to the study website (www.imaginestudy.org).

We assessed eligibility using an online prescreening form. Additional in‐session screening included smoking assessment, a record of medication, measures of effect by the Depression Anxiety and Stress Scale 21 (DASS‐21; Anthony et al. 1998), mental health history by the Mini‐International Neuropsychiatric Interview (MINI; Sheehan et al. 1997), history of substance abuse/dependence by the Alcohol Use Disorders Identification Test (AUDIT; Saunders et al. 1993), and the Cannabis Use Disorder Identification Test‐Revised (CUDIT‐R; Adamson et al. 2010). See Supporting Information S1 for screening measures details.

Exclusion criteria for both groups were self‐reported neurological impairment, learning disability, current psychotic symptoms that would impair performance, substance abuse/dependence, current regular smoking, and current high risk of suicide. Participants with a BMI < 18.5 (underweight) were excluded from both groups. Exclusion criteria for the HC group were a history of mental health issues/diagnoses and DASS‐21 scores outside the normal range (Anthony et al. 1998). B/P participants were included if they experienced ≥ 1 episode of binging and/or purging per week over the previous 4 weeks, as per DSM‐5 diagnostic criteria of frequency and duration. We therefore aimed to include individuals with threshold and subthreshold ED, including participants with BN, AN‐R, and AN‐B/P. However, in alignment with our aim of characterizing ABs in dysregulated eating behavior, a formal diagnosis of any ED was not required at screening.

### 2.2 Procedure

All participants gave informed consent prior to testing. Experimental sessions were conducted online via Zoom or Microsoft Teams and lasted 2.5–3.5 h. Following the testing session, participants were reimbursed for their time (£40–£50).

### 2.3 Measures

Self‐reported measures collected during the testing session were the McLean Screening Instrument for Borderline Personality Disorder (MSI‐BPD; Zanarini et al. 2003), hunger assessment by the Power of Food (PFS; Lowe et al. 2009), Eating Disorders Examination Questionnaire (EDE‐Q; Fairburn and Beglin 1994), Positive and Negative Affect Scale (PANAS; Watson et al. 1988), Need‐Threat Scale (NTS; Williams 2009), IQ assessment by the National Adult Reading Test [NART; Nelson and Wilson, 1991]), and BMI. See Supporting Information S1 for full questionnaires’ description.

### 2.4 Dot‐Probe Task

**FIGURE 1:**
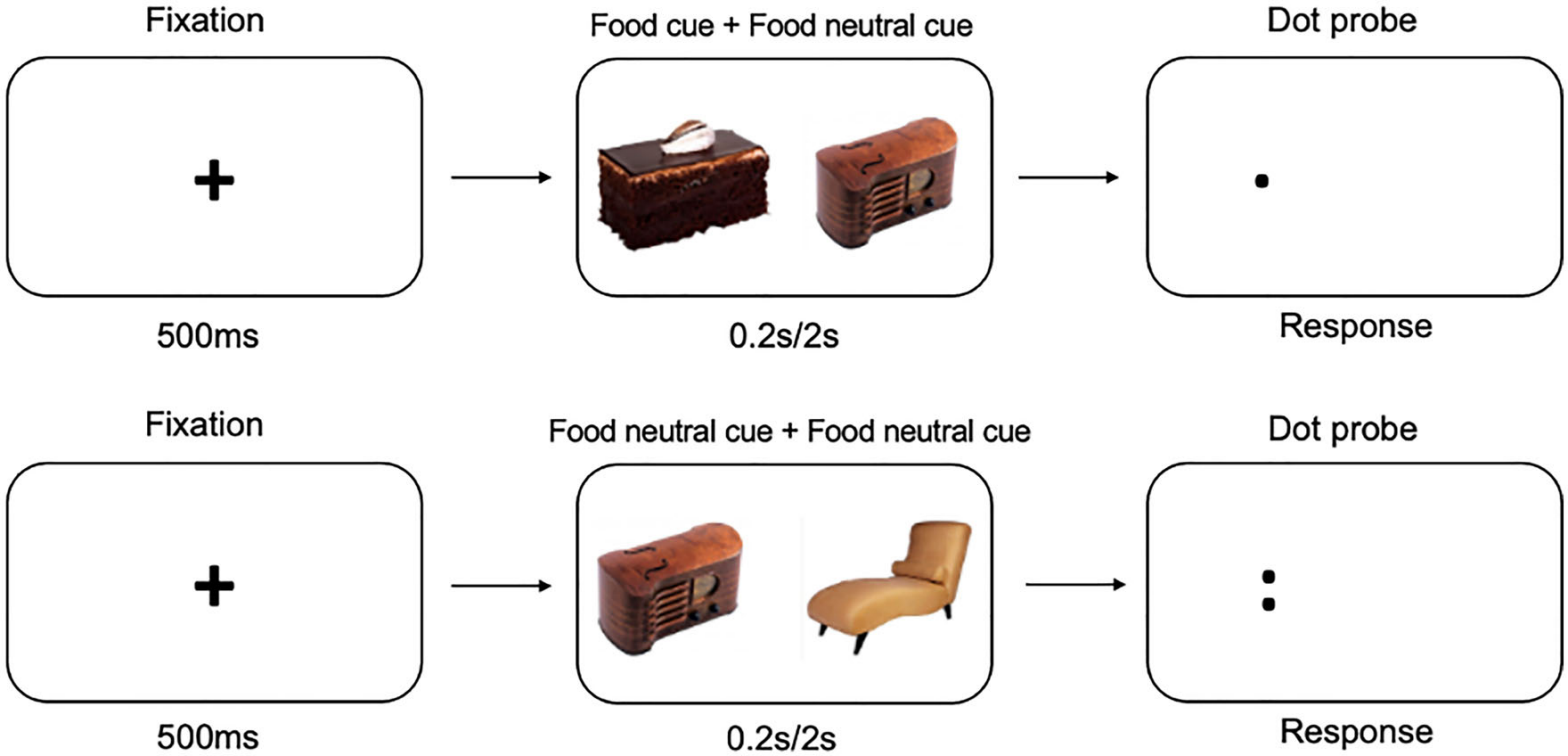
Dot‐probe task design and stimuli showing a congruent trial (top) and a neutral trial (bottom).

AB toward food and body shape cues was measured using a pictorial dot‐probe task (modified from Constantinou et al. 2010) (Figure 1) created using Psychopy (version 2021.2.3) and made available online via www.pavlovia.org. Stimuli were photographs of food, body shape, and neutral objects matched for color, shape, and complexity. Food stimuli depicted high‐calorie foods, and body shape stimuli showed normal‐weight, overweight bodies or body parts.

After presenting a fixation cross for 500 ms, randomized pairs of pictorial stimuli were presented on the left and right of the screen for either 0.2 or 2 s. Probe appearance (one or two dots) was counterbalanced to be once on the left and once on the right of the screen, both for 0.2 and 2 s, substituting either a salient stimulus (congruent trial) or neutral stimulus (incongruent trial). Participants needed to identify the number of dots by pressing either the “O” or “T” keys for one or two dots, respectively. Reaction times were measured. The task consisted of a practice round followed by 4 blocks of 80 trials, each with 20 food trials, 20 body shape trials and 40 neutral trials in each block.

The task design assumed that disorder‐relevant stimuli would capture attention more easily (AE) and make disengagement (attentional disengagement; AD) more difficult than neutral stimuli. AE and AD indexes at both 0.2 and 2 s presentation times were computed for each participant. The first three trials were excluded to ensure the participants had acclimatized to the task. Outlier trials with RT > 3 s and < 0.3 s were excluded based on Price et al. (2015). Each index was calculated in the following way:

$$\begin{array}{ccc} \mathrm{Attentional}\;\mathrm{Engagement}\;\mathrm{Index} & = & \left(\mathrm{mean}\;\mathrm{RT}\;\mathrm{incongruent}\;\mathrm{trial}\right)\\ & & -\left(\mathrm{mean}\;\mathrm{RT}\;\mathrm{congruent}\;\mathrm{trial}\right) \end{array}$$

$$\begin{array}{ccc} \mathrm{Attentional}\;\mathrm{Disengagement}\;\mathrm{Index} & = & \left(\mathrm{mean}\;\mathrm{RT}\;\mathrm{incongruent}\;\mathrm{trial}\right)\\ & & -\left(\mathrm{mean}\;\mathrm{RT}\;\mathrm{neutral}\;\mathrm{trial}\right) \end{array}$$

### 2.5 Cyberball Task

In Study 2, the Cyberball task (Williams et al. 2000; Scheithauer et al. 2013), a computerized ball‐tossing game, was used to initiate social exclusion. Participants were instructed to play online with two researchers they had previously engaged with in a separate stress task in the testing session, although the other players were computer‐generated avatars. The game was designed with EmpiriSoft and accessed via Qualtrics XM. There were four rounds of the game, which lasted approximately 5 min. The first practice computer‐controlled round consisted of 10 throws. In the second and third inclusion rounds, participants received 10 tosses over 30 total throws (33.33%). In the fourth exclusion round, participants received 5‐ball tosses over 60 total throws (8.33%). PANAS and NTS questionnaires were administered before and after the Cyberball task and after the dot‐probe task to check whether exclusion effects were maintained after the task.

### 2.6 Statistical Analysis

A one‐way ANOVA power calculation using data from Constantinou et al. (2010) comparing ABs of current opiate users with ex‐users and HCs in the dot‐probe task indicated that for an effect size of *f* = 0.382 at 80% power, *N* = 24 was needed per group for the 0.2 s condition, and for an effect size of *f* = 0.259 at 80% power, *N* = 49 were needed per group for the 2 s condition.

All data were analyzed using IBM SPSS Statistics 28.0.0.0 (190). For both studies, participants’ demographical and clinical characteristics were compared using independent samples *t*‐tests or chi‐square tests when appropriate. For both studies, two separate 2 (type of cue: food vs. body shape) × 2 (group: B/P vs. HC) mixed‐design ANOVAs were used to investigate AE and AD at 0.2 and 2 s. Independent samples *t*‐tests were performed on the accuracy (% of correct trials).

For Study 2, three separate 3(time: pre‐Cyberball, post‐Cyberball, post‐dot‐probe) × 2(group: B/P vs. HC) mixed‐design ANOVAs for positive PANAS, negative PANAS, and NTS subscales scores were used to test the efficacy of the Cyberball task. Bonferroni‐corrected post hoc comparisons were performed on positive and negative PANAS scores. For both studies, we explored associations between measures of AB, affect (negative PANAS, NTS, DASS‐21 total, and stress, anxiety, and depression subscale scores) and ED psychopathology (EDE‐Q) using Pearson's correlations. As these correlations were exploratory, no correction for multiple comparisons was applied.

## 3 Results

### 3.1 Samples

See Table 1 for participants’ characteristics. Our B/P sample consisted of individuals who self‐reported binge or purge behavior, that is, with threshold and subthreshold ED, including participants with BN, AN‐R, and AN‐B/P (see Table 2 for B/P group clinical characteristics).

**TABLE 1:** Participants’ characteristics.

	Study 1			Study 2		
	HC (*N* = 54)	B/P (*N* = 53)	*p*	HC (*N* = 73)	B/P (*N* = 72)	*p*
Age (M ± SD)	19.45 ± 2.41	19.27 ± 2.82	NS	20.11 ± 2.70	19.74 ± 2.73	NS
Gender (F: M: NB: GL: Ns)	38: 6: 0: 0: 10	43: 4: 4: 0: 2	0.018	60: 12: 0: 0: 1	65: 3: 2: 2: 0	0.032
Ethnicity White Asian Black/African/Caribbean Mixed/Multiple Ethnic Groups Other Not specified	14 18 2 2 8 0	28 12 4 5 2 0	0.005	23 35 5 5 5 0	28 20 10 10 1 3	NS
IQ (M ± SD)	13.87 ± 5.63	17.70 ± 6.16	0.002	9.57 ± 7.31	9.00 ± 7.35	NS
BMI (M ± SD)	22.65 ± 4.04	23.97 ± 4.70	NS	21.26 ± 3.13	22.68 ± 5.37	0.006
Restraint (M ± SD) Eating concern (M ± SD) Shape concern (M ± SD) Global (M ± SD)	(0.61 ± 0.93) (0.30 ± 0.65) (1.04 ± 1.13) (0.701 ± 0.83)	(2.32 ± 1.49) (3.18 ± 1.58) (3.61 ± 1.41) (3.24 ± 1.37)	< 0.001 < 0.001 < 0.001 < 0.001	(0.69 ± 0.92) (0.33 ± 0.57) (0.99 ± 1.01) (0.73 ± 0.68)	(3.33 ± 1.70) (3.42 ± 1.48) (4.65 ± 1.32) (3.93 ± 1.32)	< 0.001 < 0.001 0.032 < 0.001
Anxiety (M ± SD) Depression (M ± SD) Stress (M ± SD)	(2.47 ± 2.39) (1.39 ± 1.73) (4.18 ± 3.23)	(20.81 ± 11.08) (16.61 ± 10.52) (23.54 ± 10.89)	< 0.001 < 0.001 < 0.001	(1.89 ± 2.23) (1.89 ± 2.08) (4.08 ± 3.78)	(19.94 ± 10.90) (23.67 ± 10.50) (25.50 ± 9.97)	< 0.001 < 0.001 < 0.001
Power food scale Available Present Tasted	(1.61 ± 0.64) (1.80 ± 0.62) (2.06 ± 0.69)	(3.63 ± 1.01) (3.87 ± 1.11) (3.06 ± 0.91)	< 0.001 < 0.001 < 0.001	(1.87 ± 0.77) (2.09 ± 0.76) (2.39 ± 0.83)	(3.76 ± 0.81) (3.86 ± 0.91) (3.10 ± 1.00)	< 0.001 < 0.001 < 0.001

*Note*. Demographics were collected for all participants who completed the dot‐probe task. IQ refers to NART total errors (higher number of errors means lower IQ); non‐native English speakers and participants with dyslexia were excluded from reported means.

Abbreviations: BMI = body mass index (kg/m^2^); F = female; GL = gender fluid; IQ = Intelligence Quotient; M = male; M = mean; NB = non‐binary; NS = not significant; Ns = not specified; SD = standard deviation.

**TABLE 2:** B/P group clinical characteristics.

		Study 1	Study 2	
		B/P (*N* = 53)	B/P (*N* = 72)	
AUDIT (M ± SD)		6.25 ± 6.08	5.75 ± 6.34	
CUDIT (M ± SD)		1.39 ± 4.32	0.65 ± 0.84	
MSI‐BPD (Y:N:U)		24:26:3	30:36:4	
MINI Neuropsychiatric Interview Major depression (current:recurrent) Dysthymia current Suicide risk (low:moderate:high) Hypomanic episode (current:past) Manic episode (current:past) Panic episode (current:past) Panic disorder limited symptoms lifetime Agoraphobia (current) Social phobia (current) OCD (current) PTSD (current) Alcohol use current (abuse:dependence) Substance use current (abuse:dependence) Mood disorder with psychotic features (current:past) Psychotic disorder (current:past) Bulimia Nervosa (current) Anorexia Nervosa (current) Anorexia binge‐purge type (current) Generalized anxiety (current)		22:8 2 7:8:7 1:3 0:6 7:12 7 13 19 11 3 0:3 0:3 2:5 1:3 41 0 0 20	43:24 37 11:10:7 9:3 5:7 29:0 14 31 26 29 10 0:2 0:1 2:0 4:0 59 1 3 34	
Current psychotropic medication Atypical antidepressant Atypical antipsychotic Anticonvulsant Benzodiazepine Serotonin Reuptake inhibitor (SSRI) Serotonin‐norepinephrine Reuptake Inhibitor (SNRI) Stimulant Triptan Tricyclic antidepressant	1 3 1 0 5 1 1 1 1			1 6 1 4 13 2 0 0 0
McLean Screening Instrument for Borderline Personality Disorder (MSI‐BPD) (present:unknown)	0:1			30:4

*Note*: Clinical characteristics were collected for all participants who completed the dot‐probe task.

Abbreviations: M = mean; N = no; OCD = obsessive‐compulsive disorder; PTSD = post‐traumatic stress disorder; SD = standard deviation; SNRI = serotonin‐norepinephrine reuptake Inhibitor; SSRI = serotonin reuptake inhibitor; U = unknown; Y = yes.

### 3.2 Study 1

#### 3.2.1 Dot‐Probe Task

Outliers in task performance (*N* = 1 HC and *N* = 6 B/P) were excluded according to box‐plot norms (Price et al. 2015) and unusual observations in testing session notes (e.g., distraction, interruption, slow internet).

An independent samples *t*‐test indicated that participants with B/Ps (M_B/P_ = 93.453, SD_B/P_ **= 9.848) were significantly less accurate in responding to the probe than HCs (M_HC _= 97.214, SD_HC_ = 2.792) (*t*[105] = 2.698, *p* = 0.008, *d* = 0.522) across the task.

**FIGURE 2:**
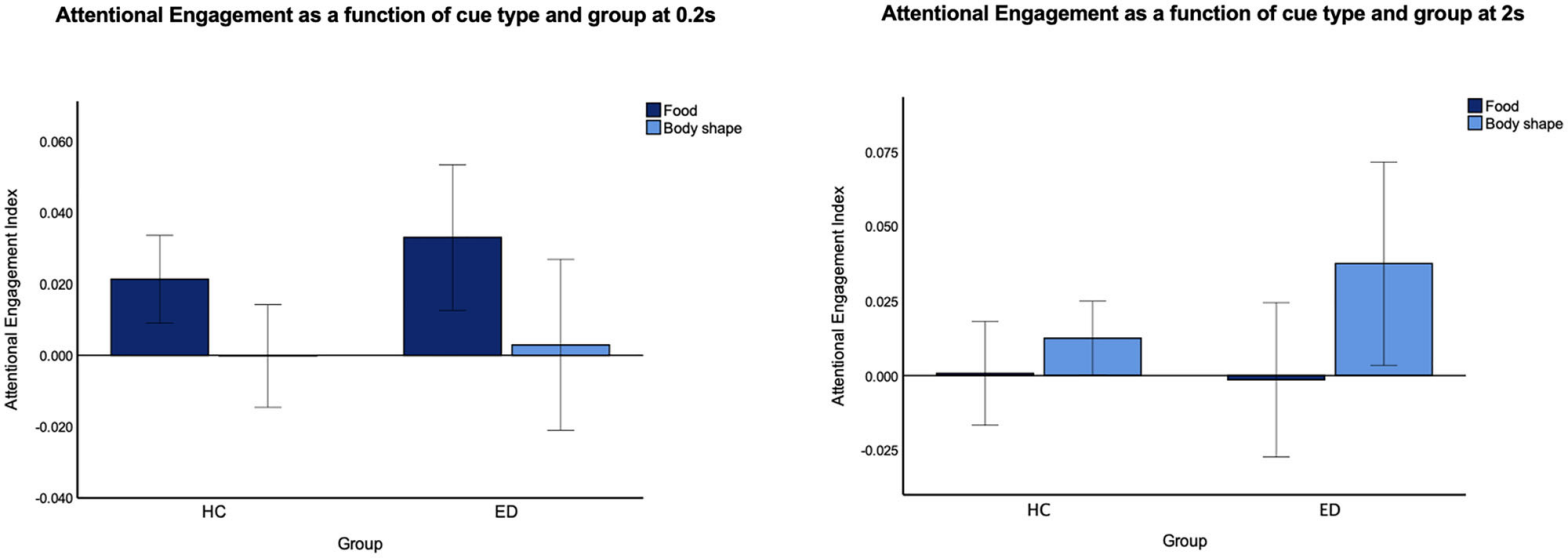
Clustered bar graph showing mean AE indexes at 0.2 s (left) and 2 s (right) as a function of cue type and group. Error bars denote 95% CI.

There was a significant main effect of cue type on AE at 0.2s ($\eta_{p}^{2}$ = 0.075) but no main effect of group. Inspection of the means suggested that all participants experienced more AE toward food ($M_{\mathrm{food}}$ = 0.027, $SD_{\mathrm{food}}$= 0.057) than body shape cues ($M_{\mathrm{shape}}$ = 0.001, $SD_{\mathrm{shape}}$ = 0.067). There was a significant main effect of cue type on AE at 2 s ($\eta_{p}^{2}$ = 0.043), but no significant main effect of group. Inspection of the means suggested that all participants showed more AE toward body shape rather than food cues ($M_{\mathrm{shape}}$ = 0.024, $SD_{\mathrm{shape}}$ = 0.087; $M_{\mathrm{food}}$ = −0.000, $SD_{\mathrm{food}}$ = 0.075). There were no significant group by cue type interactions at either 0.2 s or 2 s. See Figure 2 for a graphical representation of the AE analyses. See Table S2 for descriptive statistics and Table S4 for full AE analyses.

**FIGURE 3:**
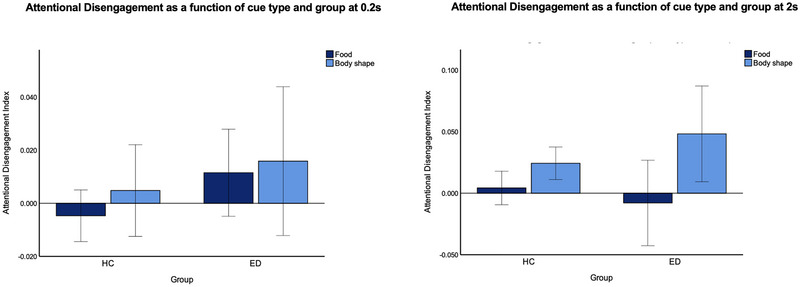
Clustered bar graph showing mean AD indexes at 0.2 s (left) and 2 s (right) as a function of cue type and group. Error bars denote 95% CI.

As for AD at 0.2 s, no significant main effect of type of cue group and no interaction between group and cue type were found. At 2 s, there was a significant main effect of type of cue on AD with a medium effect size, where all participants found it harder to disengage from shape ($M_{\mathrm{shape}}$ = 0.035, $SD_{\mathrm{shape}}$ = 0.098) rather than food cues ($M_{\mathrm{food}}$ = −0.002, $SD_{\mathrm{food}}$ = 0.088). No significant main effect of group and no interaction between group and cue type were found. See Figure 3 for a graphical representation of the AD analyses. See Table S3 for descriptive statistics and Table S5 for full AD analyses. ANCOVA with age, IQ, and BMI as covariates was also run (see Tables S6 and S7).

Correlation analysis performed for the B/P group on AB measures revealed a significant positive relationship between AE toward food cues at 0.2 s and EDE‐Q restraint (*r* = 0.323, *p* = 0.019) and between AE toward food cues at 2 s and DASS‐21 stress scores (*r* = 0.353, *p* = 0.010). See Table S8 and Figures S1 and S2 for full correlation analyses.

### 3.3 Study 2

#### 3.3.1 Mood Manipulation

**FIGURE 4:**
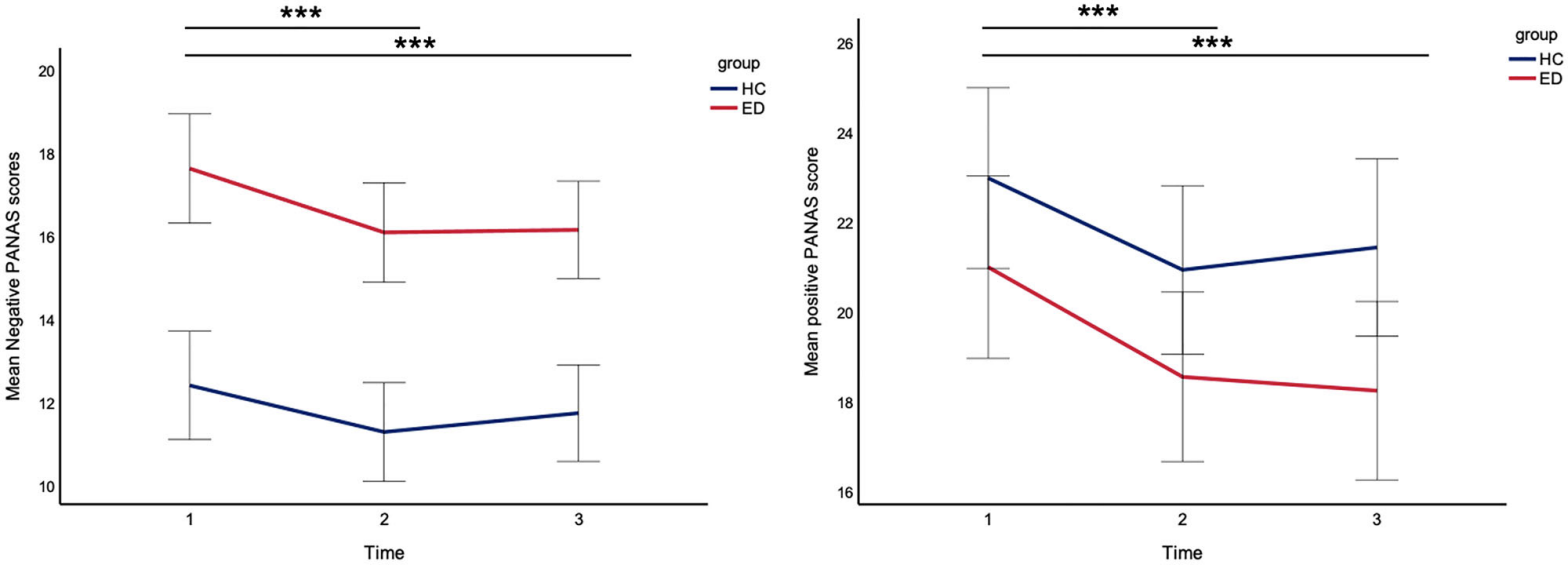
Line graph showing mean negative (left) and positive (right) PANAS scores as a function of group at different time points. Error bars denote 95% CI. Time 1, pre‐Cyberball; Time 2, post‐Cyberball; Time 3, post‐dot‐probe. ***significance at 0.001.

The mixed ANOVA on negative PANAS scores revealed a significant main effect of time (*F*[2,264] = 9.943, *p *< 0.001, $\eta_{p}^{2}$ = 0.070) and group (*F*[1,132] = 36.020, *p *< 0.001, $\eta_{p}^{2}$ = 0.214), with participants with B/Ps presenting higher mean NA scores compared to HCs. No significant interaction between time and group was present. Post hoc analysis exploring the main effect of time indicated that negative PANAS scores reduced significantly from before to after the Cyberball task and after the dot‐probe task (Figure 4).

There was a significant main effect of time (*F*[2,264] = 11.040, *p *< 0.001, $\eta_{p}^{2}$ = 0.077) and group (*F*[1,132] = 4.989, *p *< 0.001, $\eta_{p}^{2}$ = 0.036) on positive PANAS scores, with higher average values for HCs compared to participants with B/Ps across time points. No significant interaction between time and group was found. A Bonferroni‐corrected post hoc comparison indicated that positive PANAS scores reduced significantly from before to after the Cyberball task and after the dot‐probe task but remained stable between the Cyberball task and after the dot‐probe task. See Tables S9 and S10 for full statistical analyses on the PANAS. See Tables S11 and S12 for statistical analyses on NTS scores measured before and after the dot‐probe task.

#### 3.3.2 Dot‐Probe Task

Outliers in task performance (*N* = 5 HC and *N* = 5 B/P) were excluded based on Price et al. (2015).

An independent samples *t*‐test performed on accuracy indicated no significant difference between the HC (M_HC_ = 95.867, SD_HC_ = 2.759) and B/P groups (M_B/P_ = 95.188, SD_B/P_ = 3.926) (*t*[133] = 1.163, *p* = 0.247, *d* = 0.200) across the task.

No main effect of cue type and group and no significant interaction between cue type and group were found for AE and AD at both 0.2 s and 2 s. See Tables S13–S16 for full statistical analyses on AE and AD and Figures S3–S6 for graphical representations. ANCOVA with age, IQ, and BMI as covariates was also run (see Supplementary Tables S17 and S18).

Correlation analysis performed for the B/P group revealed a significant relationship between AE toward body shape cues at 0.2s and DASS‐21 anxiety (*r* = 0.301, *p* = 0.010), depression (*r* = 0.367, *p* = 0.002), stress (*r* = 0.233, *p* = 0.049), EDE‐Q shape concern (*r* = 0.246, *p* = 0.038) and PFS food available (*r* = 0.279, *p* = 0.019). At 2 s, AE toward food cues correlated with stress (*r* = −0.235, *p* = 0.047), and AE toward body shape cues correlated with PFS food available (*r* = −0.290, *p* = 0.014). Significant correlations were also found between AD to food cues at 0.2 s and DASS‐21 anxiety (*r* = 0.241, *p* = 0.042), and between AD to body shape cues at 0.2 s and both DASS‐21 anxiety (*r* = 0.272, *p* = 0.021) and depression (*r* = 0.314, *p* = 0.007). At 2 s, AD from food cues correlated with EDE‐Q eating concern (*r* = 0.241, *p* = 0.043) and shape concern (*r* = 0.235, *p* = 0.049), whereas AD from shape cues correlated with PFS food available (*r* = 0.341, *p* = 0.004). No other significant correlations were found between eating disorder psychopathology and AB measures. See Table S19 and Figures S7–S19 for all correlations.

## 4 Discussion

This study aimed to characterize ABs in transdiagnostic dysregulated eating behaviors in young people. Contrary to our hypotheses, we did not find any evidence of ABs toward food or body shape cues in a transdiagnostic sample of young people with disordered eating compared to age‐matched controls who did not report binging or purging behavior.

Our findings contradict previous dot‐probe studies that identified ABs toward food and body shape cues specific to young people who B/P (Rieger et al. 1998; Shafran et al. 2007; Blechert et al. 2010). However, these studies all differed in sample size and characteristics, and task design. For example, two studies compared a similar number of individuals diagnosed with BN and AN to HCs, respectively, using words (Rieger et al. 1998) and pictorial body shape stimuli (Blechert et al. 2010). Another study used photos of food, body shape, and weight in a small cohort of individuals with disturbed eating compared to HCs and confirmed the presence of Abs toward food but not body shape stimuli in a follow‐up study with a larger sample of adult female participants (Shafran et al. 2007).

Another consideration is that ABs noted in previous research have been shown to disappear with increased stimulus presentation times, from 500 to 2000 ms (Lee and Shafran 2008). Stimulus presentation times differ across all dot‐probe studies, including ours, and it is possible that our choice of stimulus presentation times (200 and 2000 ms) might have been too short and too long, respectively, to capture differences in ABs between groups (Shafran et al. 2007; Lee and Shafran 2008; Meyer et al. 2000).

In addition, previous studies did not find ABs toward food in ED samples compared with HCs using highly palatable food cues (Shank et al. 2015; Deluchi et al. 2017), as these could elicit hunger‐motivated ABs regardless of psychopathology (Castellanos et al. 2009; Nijs et al. 2010). Moreover, the presentation of images of both low‐ and high‐caloric foods might not be able to capture psychopathology‐related bias within a mixed binge‐purge‐restricting sample with heterogeneous approach‐avoidance tendencies to food cues (Ahern et al. 2010). Similarly, as differences in body shape perception are present between ED diagnoses (Gailledrat et al. 2016), the presentation of both normal weight and overweight body images in our study might have masked the presence of ABs toward body shape in our samples.

In Study 2, findings of ABs across both HC and B/P groups were not replicated, and contrary to previous evidence, no indication of B/P‐specific ABs toward body and shape cues was found after NA induction (Smith et al. 2020). Cross‐task inconsistencies could be due to within‐subject variability in attentional processing (Iacoviello et al. 2014; Price et al. 2015; Rodebaugh et al. 2016) and small sample size (Hampshire et al. 2012). It is possible that our design choice of the Cyberball task (e.g., task length, number of exclusion trials) may have resulted in an insufficient exclusion manipulation, and we also did not measure the effects of social exclusion throughout all stages of the game (i.e., comparing inclusion vs. exclusion rounds) (Dewald‐Kaufmann et al. 2021). However, our B/P sample had a higher need‐threat compared to HCs (Cardi et al. 2019; Meneguzzo et al. 2020), comparable to those observed in a study of patients with AN (Meneguzzo et al. 2020).

As expected, participants with B/P generally presented higher NA and lower positive affect (PA) scores compared to the HC group. Positive PANAS decreased post‐Cyberball across groups. However, contrary to our hypothesis, negative PANAS also decreased, which could be due to participants feeling more relaxed once starting the task compared to their prior expectations. Overall, the Cyberball task might not have been successful at evoking negative emotions. Nevertheless, past research has shown that social exclusion might not be effective in causing affective changes (Blackhart et al. 2009; Gerber and Wheeler 2009; Twenge et al. 2003) due to participants’ attempts at emotional regulation, such as delayed moderation of need satisfaction (Williams et al. 2009; Hartgerink et al. 2015) and efforts for re‐inclusion (Dewald‐Kaufmann et al. 2021). Higher accuracy in the B/P group post‐Cyberball compared to Study 1 might indicate participants’ redemptive attempt to perform better in the task (Jamieson et al. 2010).

On the other hand, research also suggests that the consequences of negative interpersonal experiences on eating behavior might not be mediated by changes in affective states (Baumeister 2005; Javaras et al. 2022; Oaten 2008; Rieger et al. 2010). Momentary changes in PA, but not in NA, have been shown to interact with eating expectancies (Hardman et al. 2014) and ABs toward palatable foods (Vogt et al. 2011) in binge eating (Smith et al. 2020). However, it has also been argued that PA might not be an accurate predictor of future eating behavior (Crawford et al. 2004; Merz et al. 2011; Dingemans et al. 2009). Hence, while our conclusions are not consistent with previous literature indicating greater ABs to both foods (Blechert et al. 2014; Hepworth et al. 2010; Rofey et al. 2004) and body shape (Allen et al. 2018) cues following affect manipulation, it is possible that changes in affect alone might not be enough to elicit ABs in young people who B/P.

Our exploratory findings suggest that ABs might be correlated with general psychopathology and B/P symptomatology, more evidently following mood induction and with a larger sample. Within an incentive‐sensitization theory, it is possible that disordered eating becomes conditioned over time. Therefore, ABs to food and body shape cues might be more relevant in clinical populations compared to a heterogeneous nonclinical sample of young people, including both diagnosable and subthreshold psychopathology.

Our results suggest that disordered eating, in particular B/P behavior in young people, is not associated with ABs toward food/body shape stimuli when compared to HCs, including after a social exclusion induction. These results are consistent with findings from the same studies showing the absence of motivational biases in the same population (manuscript in preparation). ABs might also not be detected by simple behavioral markers, and psychophysiological measures might be required (Blechert et al. 2011; Leehr et al. 2018; Mohr et al. 2010). It is also possible that incentive sensitization might not occur in young people with subclinical B/P symptomatology measured transdiagnostically and/or that different reinforcement profiles might underline binging and purging behaviors.

### 4.1 Limitations

This is the first study examining ABs and their association with NA in a relatively large transdiagnostic sample of young people with binging/purging behavior. However, we did not adjust our analysis based on ratings of hunger or satiety (Nijs et al. 2010) which is likely to be especially relevant for AB toward food cues. The use of psychotropic medication, which could impact cognitive performance and reward processing (Zhang et al. 2020; McCabe et al. 2010), was also uncontrolled. Hormonal factors such as the menstrual cycle stage might have also influenced our findings (Krishnan et al. 2016). Due to the large amount of missing data for BMI, its confounding effect was also not included in the main analysis. Moreover, while our predominantly white female sample represents the reality of cases found in clinical practice, findings might differ in a more diverse population.

As for limitations of our tasks design, the inclusion of two types of stimuli in the dot‐probe task led to having fewer trials per cue type, which might have diminished the power of our analyses. Online testing prevented control of the experimental setting, so despite excluding performance outliers, participants might have been more distracted and/or felt more comfortable in their own environment. Online testing might have also introduced more variability to RTs due to differing internet speeds, computers, etc. Moreover, the Cyberball task might have been too short and/or not contain enough exclusion trials to induce significant feelings of social exclusion.

### 4.2 Future Directions

The adoption of a more homogeneous approach to the study of ABs could allow for clarifying some of the literature's inconsistencies, for example, standardizing stimuli presentation latencies, type of cues, and tailoring cues to individuals’ psychopathology. Future research should also consider different mood manipulations and analyze differences between social and nonsocial stressors. Recent research has begun to focus on social attention biases as having specific salience in subjects with EDs (Rowlands et al. 2020). Different studies have confirmed the validity of more direct measures of ABs in eliminating some of the experimental shortcomings of RT‐based tasks, including eye‐tracking (Leehr et al. 2018), event‐related potentials (ERP) (Blechert et al. 2011), and functional magnetic resonance imaging (fMRI) (Mohr et al. 2010). The use of psychophysiological indices of implicit ABs would help to elucidate the role of ABs as a cognitive marker in B/Ps. Whether ABs are gender‐specific and the extent to which they vary by BMI also needs additional exploration.

In conclusion, based on our findings, ABs related to food and body shape do not seem to be a viable treatment target transdiagnostically in the treatment of ED behavior. Notwithstanding the study design limitations, other alterations in cognitive domains, such as cognitive flexibility, inhibitory control, and decision‐making, need investigating as potential targets for interventions that could be easily scalable therapeutic adjuncts across diagnosis, or subclinical presentations where disordered eating is present.

## Author Contributions

R.R., V.B., and M.D.S. designed the study. R.R. was responsible for the overall organization and coordination of the study. A.F., V.B., and A.G. conducted data collection and processing. A.F. was responsible for data analysis. M.D.S. was the chief investigator and overviewed all study aspects. D.N. contributes eating disorders expertise to study design and results interpretation. All authors contributed to the writing of the final manuscript.

## Ethics Statement

The study was approved by the NRES Committee South Central Oxford‐C (REF: 19/SC/0275). The authors assert that all procedures contributing to this work comply with the ethical standards of the relevant national and institutional committees on human experimentation and with the Helsinki Declaration of 1975, as revised in 2008.

## Conflicts of Interest

The authors declare no conflicts of interest.

### Peer Review

The peer review history for this article is available at https://publons.com/publon/10.1002/brb3.70322.

## Supporting information



Supporting Information

## Data Availability

The data that support the findings of this study are openly available in “iMAGine: Motivational Abnormalities Guiding self‐harm and binge eating” at https://osf.io/jpnxh/, reference number DOI 10.17605/OSF.IO/JPNXH.
