# Supplementary material for: Attentional Processing Biases in Young People With Binging and Purging Behavior
Source: Brain Behav. 2025 Feb 16;15(2):e70322. doi: 10.1002/brb3.70322 (PMC11830632; doi:10.1002/brb3.70322)
Supplement: Supplementary file 1 — Supporting Information [file BRB3-15-e70322-s001.docx]

**Supplementary materials**

**Attentional processing biases in young people with binging and purging behaviour**

Authors: Freccero A^1^, Burmester V^1^, Rodrigues R^1^, Gallucci A^2^, Nicholls D^1^, Di Simplicio M^1^

1. Division of Psychiatry, Department of Brain Sciences, Imperial College London, United Kingdom
2. IRCCS Fondazione Don Carlo Gnocchi, Milan, Italy

**S1 Assessments and Questionnaires**

Mental Health

*MINI International Neuropsychiatric Interview*. The MINI (Sheehan et al., 1997) is a short structured diagnostic interview employed for the detection of the most common psychiatric disorders compatible with international diagnostic criteria (i.e. ICD-10, DSM-5).

*McLean Screening Instrument for Borderline Personality Disorder (MSI-BPD*). MSI-BPD (Zanarini et al., 2003) is a validated 10-item screening measure for Borderline Personality Disorder (BPD) with all items based on the DSM-1V diagnostic criteria for BPD. Two items are based on the ninth DSM-IV criterion of paranoia/dissociation, and each of the remaining eight items is based on the remaining eight DSM-IV criteria.

Affect

*Depression Anxiety and Stress Scale 21 (DASS-21)*. The DASS-21 (Anthony et al., 1998) is a 21-item scale used to measure self-reported features of depression, anxiety and stress in both clinical and non-clinical groups. It has high internal consistency as well as temporal stability and allows for greater separation between depressive and anxious traits. Each item relates to the preceding week and is scored on a four-point Likert scale.

*Positive and Negative Affect Scale (PANAS).* The PANAS (Watson et al., 1988) presents a 10-item two-factor model of affective state (Positive Affect and Negative Affect) of positive and negative emotionality. Participants report on a 5-point Likert scale the extent to which they experienced different feelings and emotions at the present moment. The scales have shown high discriminant validity and internal consistency.

*Need-Threat Scale (NTS)*. The NTS (Zadro et al., 2004; Williams, 2009) is an ostracism manipulation check measuring need-threat across four fundamental needs (belonging, self-esteem, control, and meaningful existence). Our 20-item version was taken from Jamieson et al., 2010. Participants are asked to rate the extent to which they felt excluded during the Cyberball task (Williams et al., 2000) on a 5-point scale. Lower scores index less need satisfaction (i.e. more need threat).

Eating disorder symptomatology

*Eating Disorders Examination Questionnaire (EDE-Q)*. The EDE-Q version 17.0D (Fairburn and Beglin, 1994) is a self-reported measure of eating disorder psychopathology (in accordance with the DSM-V guidelines) adapted from the Eating Disorder Examination (EDE; Cooper & Fairburn, 1987; Fairburn & Cooper, 1993). It is divided into four subscales (Restraint, Eating Concern, Shape Concern, Weight Concern) reflecting the severity of several aspects of the ED symptomatology (global score) over the preceding 28-days. All items are rated on a seven-point Likert scale. It has demonstrated good test-retest reliability, internal consistency and temporal stability.

*Power of Food Scale (PFS)*. The PFS (Lowe et al., 2009) is a psychometric assessment of the psychological effects and hedonic impact of living in conditions of palatable food abundance and evaluates individuals’sensitivity to food reward in three domains of food proximity (food available, food present and food tasted) (Cappelleri et al., 2009).

*Craving Experience Questionnaire for Eating Disorders (CEQ-ED).* CEQ-ED is adapted from the CEQ for cigarettes and alcohol (May et al., 2014). It assesses the frequency and strength of desire (intensity) to engage in binge and purge behaviours over the past week. The subscales are frequency, assessed with 9 items, and intensity, assessed with 7 items. Items are rated using a 10-point scale, ranging from 1 “not at all” to 10 “extremely”, with a maximum score of 160. Higher scores indicate a greater frequency and strength of desire to binge and/or purge.

Based on the *Self-Injurious Thoughts and Behaviours Interview (SITBI)* (Nock et al., 2007), two items were added asking how old the participant was when they binged and/or purged for the first time, and how often they have binged and/or purged in the last year.

Substance misuse

*Alcohol Use Disorders Identification Test (AUDIT)* (Saunders et al, 1993). AUDIT is a 10-item measure of alcohol misuse and possible dependence, in which participants are asked to rate items related to their drinking (e.g. ‘How often do you have a drink containing alcohol’) on a 5-point scale. The AUDIT is an effective means of identifying hazardous or harmful drinking behaviour.

*Cannabis Use Disorder Identification Test Revised (CUDIT-R)* (Adamson et al, 2010). CUDIT-R is used to screen for problem cannabis use. Participants are asked to rate a series of eight items relating to their cannabis use (e.g. ‘How often do you use cannabis’) on a 5-point Likert scale. The measure has excellent internal consistency.

Smoking assessment:

Participants were be asked questions about their smoking and vaping, such as:

- 1. Do you smoke? Yes/No/Occasionally
  2. If yes, how many cigarettes do you smoke a day?
  3. Do you vape? Yes/No/Occasionally
  4. If yes, how many times do you vape a day?

IQ

*National Adult Reading Test (NART)*. NART (Nelson & Wilson, 1991) is a widely used measure of IQ in clinical research. Participants are given a list of 61 predefined words and asked to read these aloud. The number of words correctly and incorrectly pronounced are then scored. Higher number of NART errors means lower IQ.

Medication

Example questions about current medication:

a. Are you taking any regular medication for anxiety, depression, ADHD etc?

**Study 1**

**Table S2**. Mean and standard deviations for AE as a function of a cue type and group at presentation times 0.2s and 2s.

|  | HC (*n* = 53) | | ED (*n* = 47) | | Total (*n* = 100) | |
| --- | --- | --- | --- | --- | --- | --- |
|  | *M* | *SD* | *M* | *SD* | *M* | *SD* |
| AE 0.2s   Food  Body Shape | .021 -.000 | .044 .052 | .033 .003 | .069 .057 | .027 .001 | .057 .067 |
| AE 2s  Food  Body Shape | .001 .013 | .063 .045 | -.001 .037 | .088 .116 | -.000 .024 | .075 .087 |

*Note*. N=1 HC and N=6 ED were excluded.
AE = Attentional Engagement; HC = Healthy Control; ED = Eating Disorder

**Table S3**. Mean and standard deviations for AD as a function of cue type and group at presentation times 0.2s and 2s.

|  | HC (*n* = 53) | | ED (*n* = 47) | | Total (*n* = 100) | |
| --- | --- | --- | --- | --- | --- | --- |
|  | *M* | *SD* | *M* | *SD* | *M* | *SD* |
| AD 0.2s   Food  Body Shape | -.005  .005 | .035 .062 | .012 .016 | .056 .095 | .003 .100 | .047 .079 |
| AD 2s  Food  Body Shape | .004 .024 | .048 .047 | -.008 .048 | .118 .136 | -.002 .035 | .088 .098 |

*Note*. N=1 HC and N=6 ED were excluded.
AD = Attentional Disengagement; HC = Healthy Control; ED = Eating Disorder

**Table S4.** Output mixed-design ANOVA for AE as a function of cue type and group at presentation times 0.2s and 2s.

|  |  | AE 0.2s | | | | AE 2s | | | | |
| --- | --- | --- | --- | --- | --- | --- | --- | --- | --- | --- |
|  | | *df* | *F* | *p* | $\eta_{p}^{2}$ | *df* | *F* | *p* | $\eta_{p}^{2}$ |  |
| Between-subject effects   Group  Error (group) | | 1 97 | .727 | .396 | .007 | 1 97 | 1.099 | .297 | .011 |  |
| Within-subject effects  Cue type  Cue type x group  Error (cue type) | | 1 1 97 | 7.903 .222 | **.006** .639 | .075 .002 | 1 1 97 | 4.329 1.237 | **.040** .269 | .043 .013 |  |

*Note*. Numbers in bold denote significant values (p < 0.05).

**Table S5.** Output mixed-design ANOVA for AD as a function of cue type and group at presentation times 0.2s and 2s.

|  |  | AD 0.2s | | | | AD 2s | | | |
| --- | --- | --- | --- | --- | --- | --- | --- | --- | --- |
|  | | *df* | *F* | *p* | $\eta_{p}^{2}$ | *df* | *F* | *p* | $\eta_{p}^{2}$ |
| Between-subject effects   Group  Error (group) | | 1 97 | 2.564 | .113 | .026 | 1 97 | .221 | .640 | .002 |
| Within-subject effects  Cue type  Cue type x group  Error (cue type) | | 1 1 97 | .484 .796 | .488 .796 | .005 .001 | 1 1 97 | 8.050 1.875 | **.006** .174 | .077 .019 |

*Note*. Numbers in bold denote significant values (p < 0.05).

**Table S6.** Output mixed-design ANOVA for AE as a function of cue type and group at presentation times 0.2s and 2s adjusting for age, IQ and BMI.

|  |  | AE 0.2s | | | | AE 2s | | | | |
| --- | --- | --- | --- | --- | --- | --- | --- | --- | --- | --- |
|  | | *df* | *F* | *p* | $\eta_{p}^{2}$ | *df* | *F* | *p* | $\eta_{p}^{2}$ |  |
| Between-subject effects   age  IQ  BMI  Group  Error (group) | | 1 1 1 1 65 | 1.307  .481  .229 .704 | .257  .491  .634 .404 | .020 .007  .004 .011 | 1  1  1 1 65 | .014  3.350  1.273 .005 | .906 .072 .263 .943 | .000 .049 .019 .000 |  |
| Within-subject effects  Cue type  Cue type x age  Cue type x IQ  Cue type x BMI  Cue type x group  Error (cue type) | | 1 1  1  1 1 65 | .027 .979  4.427  .530 1.604 | .870 .326  .039  .469 .210 | .000 .015  .064  .008 .024 | 1  1  1 1 1 65 | 5.297 7.132  .404  .508 1.119 | .025 .010 .527 .479 .294 | ,075 .099 .006 .008 .017 |  |

*Note*. Numbers in bold denote significant values (p < 0.05).
AE = Attentional Engagement; HC = Healthy Control; ED = Eating Disorder; BMI = Body Mass Index

**Table S7.** Output mixed-design ANOVA for AD as a function of cue type and group at presentation times 0.2s and 2s adjusting for age, IQ and BMI.

|  |  | AD 0.2s | | | | AD 2s | | | |
| --- | --- | --- | --- | --- | --- | --- | --- | --- | --- |
|  | | *df* | *F* | *p* | $\eta_{p}^{2}$ | *df* | *F* | *p* | $\eta_{p}^{2}$ |
| Between-subject effects   age  IQ  BMI   Group  Error (group) | | 1 1 1 1 65 | .524 .561 1.019 .000 | .472 .457 .316 .999 | .008 .009 .015 .000 | 1 1  1  1 65 | 1.814 .228 1.053 .046 . | .183  .635 .309 .831 | .027  .003 .016 .001 |
| Within-subject effects  Cue type  Cue type x age  Cue type x IQ   Cue type x BMI  Cue type x group  Error (cue type) | | 1 1 1 1 1 65 | .562 .309 1.024 2.701 .386 | .456 .580 .315 .105 .536 | .009 .005 .016 .040 .006 | 1  1 1 1 1 65 | .091 .030 .463 .078 2.018 | .764 .863 .499 .781 .160 | .001 .000 .007 .001 .030 |

AD = Attentional Disengagement; HC = Healthy Control; ED = Eating Disorder; BMI = Body Mass Index

**Table S8.** Correlations table in ED sample

*Note*. Numbers in bold denote significant values. *r* and *p* values are displayed.

|  | EDE-Q | | | | PFS | | | DASS-21 | | |
| --- | --- | --- | --- | --- | --- | --- | --- | --- | --- | --- |
|  | restraint | eating concern | shape concern | global | available | present | tasted | anxiety | depression | stress |
| AE 0.2s  Food | **-.323* 0.19** | -.166 .239 | -.099 .485 | -.214 .128 | .021 .880 | .136 .333 | .062 .657 | .060 .672 | .236 .092 | .239 .087 |
| AE 0.2s  Body Shape | .014 .923 | .057 .687 | -.222 .115 | -.073 .607 | .191 .172 | .243 .080 | .141 .312 | -.224 .111 | -.235 .093 | -.186 .186 |
| AE 2s Food | .024 .865 | .169 .231 | .264 .058 | .195 .166 | .098 .487 | -.064 .650 | -.042 .766 | .133 .349 | .267 .055 | **.353* .010** |
| AE 2s Body Shape | -.074 .604 | .021 .885 | .050 723 | .011 .936 | .008 .956 | .190 .174 | -.059 .675 | .126 .374 | .181 .200 | .134 .343 |
| AD 0.2s Food | -.261 .062 | -.147 .298 | -.045 .751 | -.155 .274 | -.068 .630 | .061 .666 | -.144 .302 | -.065 .647 | .116 .412 | .164 .245 |
| AD 0.2s Body Shape | .004 .980 | -.036 .802 | -.252 .071 | -.142 .315 | .197 .158 | .197 .158 | .227 .102 | -1.62 .250 | -.186 .186 | -.109 .137 |
| AD 2s Food | -.043 .762 | .113 .427 | .234 .095 | .152 .283 | .106 .449 | -.172 .218 | -.113 .420 | .099 .487 | .231 .099 | .086 .542 |
| AD 2s Body Shape | .003 .983 | .194 .168 | .060 .672 | .094 .507 | .212 .127 | .119 .398 | .043 .761 | .052 .716 | .077 .587 | .021 .884 |
| EDE-Q restraint | - | - | - | - | **.345* .012** | -.017 .903 | -.088 .536 | .200 .155 | **.320* .021** | .186 .187 |
| EDE-Q eating concern | - | - | - | - | .221 .116 | .019 .892 | -.024 .868 | **.393** .004** | **.518** <.001** | **.424** .002** |
| EDE-Q shape concern | - | - | - | - | **.300* .031** | .008 .954 | -.070 .621 | **.338* .014** | **.394** .004** | **.298* .032** |
| EDE-Q global | - | - | - | - | .083 .603 | **.332* .032** | .012 .942 | .148 .315 | .051 .728 | .037 .806 |
| PFS available | - | - | - | - | - | - | - | .160 .257 | -.013 .929 | .047 .739 |
| PFS present | - | - | - | - | - | - | - | .102 .472 | .025 .858 | .162 .251 |
| PFS tasted | - | - | - | - | - | - | - | .238 .089 | .070 .621 | .209 .136 |

**Figure S1.** Scatterplot for correlation between AE Food 0.2s and EDE-Q Restraint (*r* = .323, *p* = .019)

**Figure S2.** Scatterplot for correlation between AE Food 0.2s and DASS-21 Stress (*r* = .353, *p* = .010)

**Study 2**

**Table S9.** Mean and standard deviations for PANAS scores as a function of group at different time points.

|  | HC (*n* = 67) | | ED (*n* = 67) | | Total (*n* = 134) | |
| --- | --- | --- | --- | --- | --- | --- |
|  | *M* | *SD* | *M* | *SD* | *M* | *SD* |
| PANAS 1   Negative  Positive | 12.39  23.10 | 2.964  8.711 | 17.49  20.93 | 6.951  7.795 | 14.94 22.01 | 5.908 8.307 |
| PANAS 2  Negative  Positive | 11.28  21.10 | 2.308  8.345 | 16.03  18.37 | 6.438  7.023 | 13.66 19.74 | 5.375 7.805 |
| PANAS 3   Negative  Positive | 11.73  21.54 | 2.863  8.624 | 16.01  18.18 | 6.104  7.463 | 13.87 19.86 | 5.213 8.209 |

*Note.* PANAS 1, pre-Cyberball; PANAS 2, post-Cyberball; PANAS 3, post dot-probe.
N=5 HC and N=5 ED were excluded for Part 2; N=1 HC did not complete the questionnaire.

**Table S10.** Output mixed-design ANOVA and post-hoc analyses for Negative and Positive PANAS scores as a function of group at different time points.

|  |  | PANAS Negative | | | | PANAS Positive | | | |
| --- | --- | --- | --- | --- | --- | --- | --- | --- | --- |
|  | | *df* | *F* | *p* | $\eta_{p}^{2}$ | *df* | *F* | *p* | $\eta_{p}^{2}$ |
| Between-subject effects   Group  Error (group) | | 1 132 | 36.02 | **<.001** | .214 | 1 132 | 4.989 | **.027** | .036 |
| Within-subject effects  Time Time x group Error (time) | | 2 2 264 | 9.943 .892 | **<.001** .411 | .070 .007 | 2 2 264 | 11.04 .586 | **<.001** .558 | .077 .004 |

*Note*. Numbers in bold denote significant values.

| Comparison  PANAS Negative | | *Mean Difference* | *SE* | | *Sig^b^* | *95% Confidence Interval^b^* | | |
| --- | --- | --- | --- | --- | --- | --- | --- | --- |
|  |  |  |  |  |  | *Lower Bound* | *Upper bound* | |
| Time 1  Time 2 | 2  3  3 | 1.284* 1.067* -.216 | .298  .327  .299 | **<.001 .004** 1.000 | | .562  .275  -.942 | 2.005 1.859 .510 |  |

*Note.* Time 1, pre-Cyberball; Time 2, post-Cyberball, time 3, post-dot-probe.

Numbers in bold denote significant values.
* Mean difference significant at the .05 level.
b. Adjustment for multiple comparisons: Bonferroni.

| Comparison PANAS Positive | | *Mean Difference* | *SE* | | *Sig^b^* | *95% Confidence Interval^b^* | | |
| --- | --- | --- | --- | --- | --- | --- | --- | --- |
|  |  |  |  |  |  | *Lower Bound* | *Upper bound* | |
| Time 1  Time 2 | 2  3  3 | 2.276* 2.157* -0.119 | .600  .576  .447 | **<.001 <.001** 1.000 | | .821  .760  -1.203 | 3.732 3.553 .964 |  |

*Note.* Time 1, pre-Cyberball; Time 2, post-Cyberball, time 3, post-dot-probe.

Numbers in bold denote significant values.
* Mean difference significant at the .05 level.
b. Adjustment for multiple comparisons: Bonferroni.

**Table S11**. Mean NTS scores as a function of group measured before and after dot-probe task

|  |  | HC (*n* = 67) | | ED (*n* = 62) | | Total (*n* = 128) | |
| --- | --- | --- | --- | --- | --- | --- | --- |
|  |  | *M* | *SD* | *M* | *SD* | *M* | *SD* |
| NTS 1   Belonging  Self-esteem  Meaningful existence  Control |  | 16.24  15.64  16.97  12.46 | 3.985  3.928  4.053  3.590 | 12.58  11.48  12.75 10.20 | 3.850 3.928  4.768 3.742 | 14.45  13.61  14.91 11.36 | 4.315  4.360  4.882 3.823 |
| NTS 2   Belonging  Self-esteem  Meaningful existence  Control |  | 17.04  15.55  17.72 13.37 | 3.496  4.035  3.930 4.519 | 14.08  11.84  13.50 11.72 | 4.199  4.416  5.354 4.188 | 15.59  13.74  15.66 12.56 | 4.119  4.602  5.120 4.422 |

*Note.* NTS 1, post-Cyberball; NTS 2, post-dot-probe.
N=5 HCs and N=5 EDs were excluded for part 2; N=3 EDs did not complete the questionnaire.

**Table S12.** Output mixed-design ANOVA for NTS scores (belonging, self-esteem, meaningful existence, control) as a function of group measured before and after dot-probe task.

|  |  |  | NTS Belonging | | | | | | | NTS Self-esteem | | | | | | | | NTS Meaningful existence | | | | | | | | NTS Control | | | | | | | |
| --- | --- | --- | --- | --- | --- | --- | --- | --- | --- | --- | --- | --- | --- | --- | --- | --- | --- | --- | --- | --- | --- | --- | --- | --- | --- | --- | --- | --- | --- | --- | --- | --- | --- |
|  |  | | *df* | *F* | | *p* | | $\eta_{p}^{2}$ | | *df* | | *F* | | *p* | | $\eta_{p}^{2}$ | | *df* | | *F* | | *p* | | $\eta_{p}^{2}$ | | *df* | | *F* | | *p* | | $\eta_{p}^{2}$ | |
| Between-subject  effects  Group  Error (group) | | | 1 129 | 30.24 | **<.001** | | .190 | | 1 129 | | 38.87 | | **<.001** | | .232 | | 1 129 | | 34.48 | | **<.001** | | .211 | | 1 129 | | 9.721 | | **.002** | | .070 | |  |
| Within-subject  effects   Time Time x group  Error (time) | | | 1 1 129 | 13.47 1.220 | | **<.001** .271 | | .095 .009 | | 1 1 129 | | .181 .502 | | .671 .480 | | .001 .004 | | 1 1 129 | | 4.807 1.000 | | **.030** .996 | | .036 .000 | | 1 1 129 | | 14.47 .900 | | **<.001** .344 | | .101 .007 | |

*Note*. Numbers in bold denote significant values.

**Table S13**. Mean and standard deviations for AE as a function of a 2(type of stimuli) x 2(group) at presentation times 0.2s and 2s following mood induction

|  | HC (*n* = 68) | | ED (*n* = 67) | | Total (*n* = 135) | |
| --- | --- | --- | --- | --- | --- | --- |
|  | *M* | *SD* | *M* | *SD* | *M* | *SD* |
| AE 0.2s   Food  Shape | .041 .012 | .086 .055 | .016 .012 | .102 .102 | .029 .012 | .095 .082 |
| AE 2s  Food  Shape | .006 .037 | .053 .163 | .020 .003 | .059 .092 | .013 .021 | .056 .133 |

N=5 HC and N=5 ED were excluded.

**Table S14.** Output mixed-design ANOVA for AE as a function of cue type and group at presentation times 0.2s and 2s following mood induction.

|  |  | AE 0.2s | | | | AE 2s | | | |
| --- | --- | --- | --- | --- | --- | --- | --- | --- | --- |
|  | | *df* | *F* | *p* | $\eta_{p}^{2}$ | *df* | *F* | *p* | $\eta_{p}^{2}$ |
| Between-subject effects   Group  Error (group) | | 1 133 | 1.297 | .257 | .010 | 1 133 | .604 | .438 | .005 |
| Within-subject effects  Cue type  Cue type x group  Error (cue type) | | 1 1 133 | 2.535 1.523 | .114 .219 | .019 .011 | 1 1 133 | .374 3.878 | .542 .051 | .003 .028 |

**Table S15**. Mean and standard deviations for AD as a function of a cue type and group at presentation times 0.2s and 2s following mood induction.

|  | HC (*n* = 68) | | ED (*n* = 67) | | Total (*n* = 135) | |
| --- | --- | --- | --- | --- | --- | --- |
|  | *M* | *SD* | *M* | *SD* | *M* | *SD* |
| AD 0.2s   Food  Body Shape | .025 .016 | .097 .046 | .007 .031 | .066 .152 | .016 .023 | .083 .112 |
| AD 2s  Food  Body Shape | .014 .029 | .042 .172 | .020 .015 | .105 .082 | .017 .022 | .080 .134 |

N=5 HC and N=5 ED were excluded

**Table S16.** Output mixed-design ANOVA for AD as a function of cue type and group at presentation times 0.2s and 2s following mood induction

|  |  | AD 0.2s | | | | AD 2s | | | |
| --- | --- | --- | --- | --- | --- | --- | --- | --- | --- |
|  | | *df* | *F* | *p* | $\eta_{p}^{2}$ | *df* | *F* | *p* | $\eta_{p}^{2}$ |
| Between-subject effects   Group  Error (group) | | 1 133 | .014 | .906 | .000 | 1 133 | .101 | .751 | .001 |
| Within-subject effects  Cue type  Cue type x group  Error (cue type) | | 1 1 133 | .343 1.926 | .559 .167 | .003 .014 | 1 1 133 | .125 .480 | .724 .490 | .001 .004 |


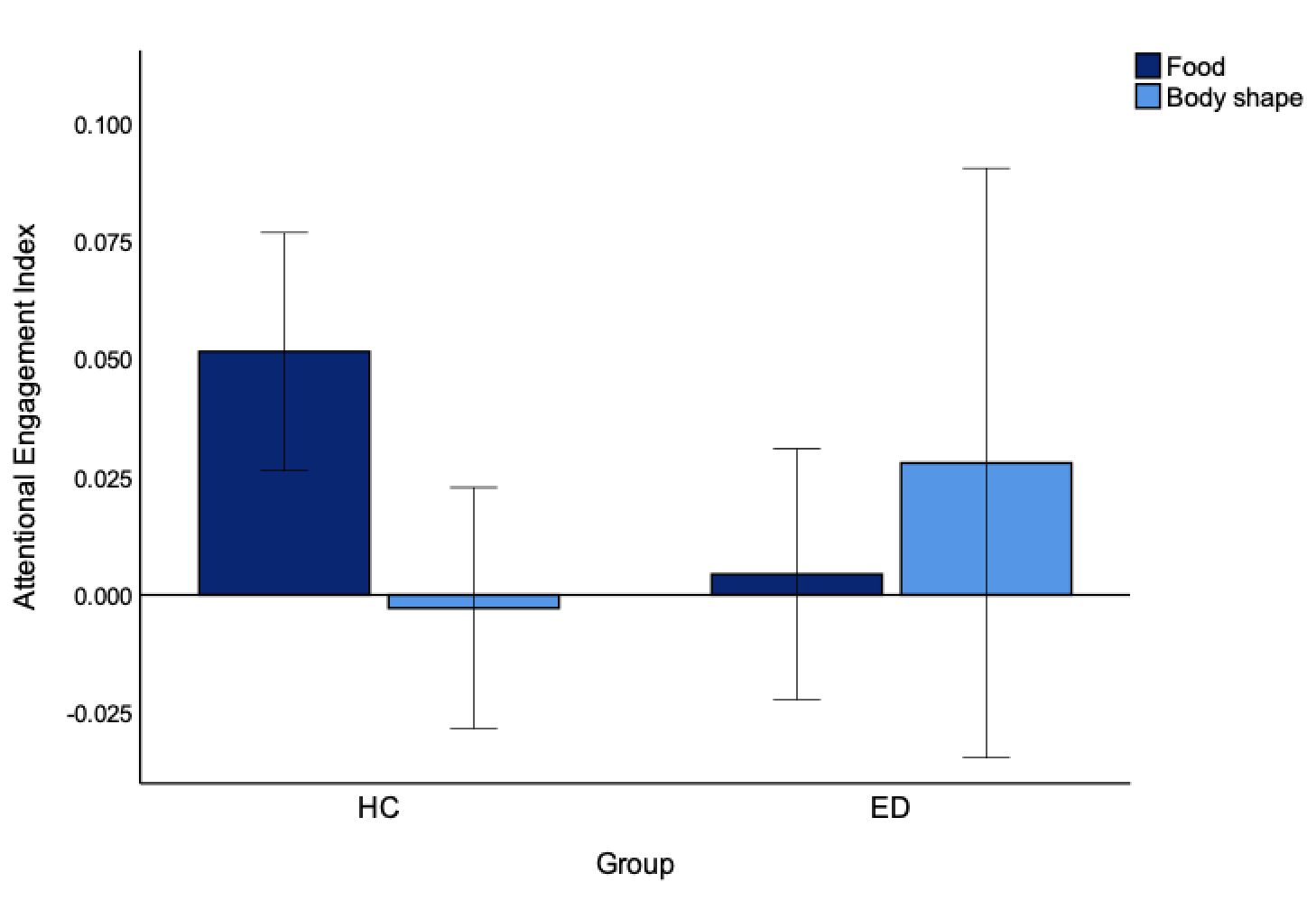


**Figure S3.** Clustered bar graph showing mean AE indexes at 0.2s as a function of cue type and group following mood induction. Error bars denote 95% CI.


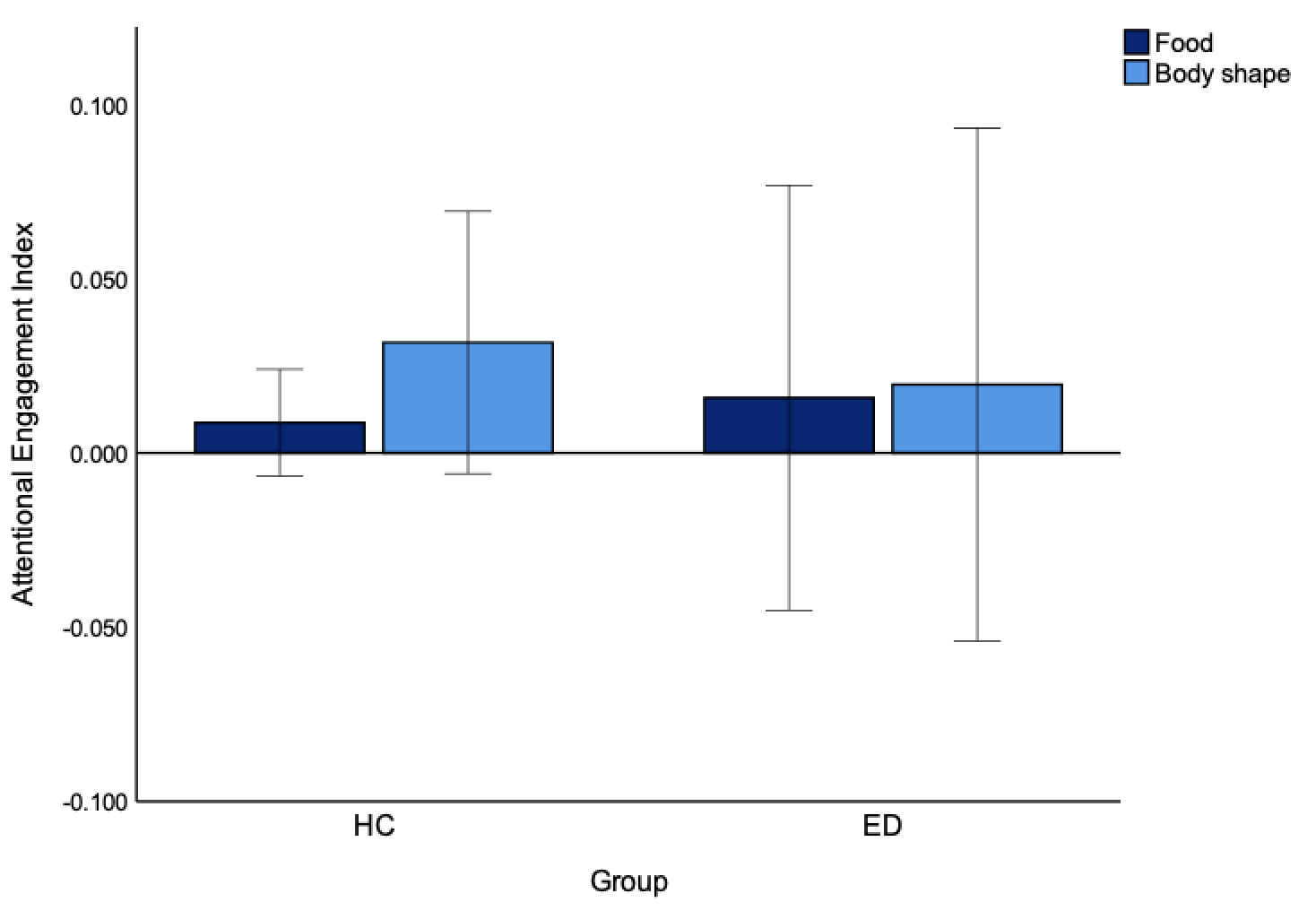


**Figure S4**. Clustered bar graph showing mean AE indexes at 2s as a function of cue type and group following mood induction. Error bars denote 95% CI.


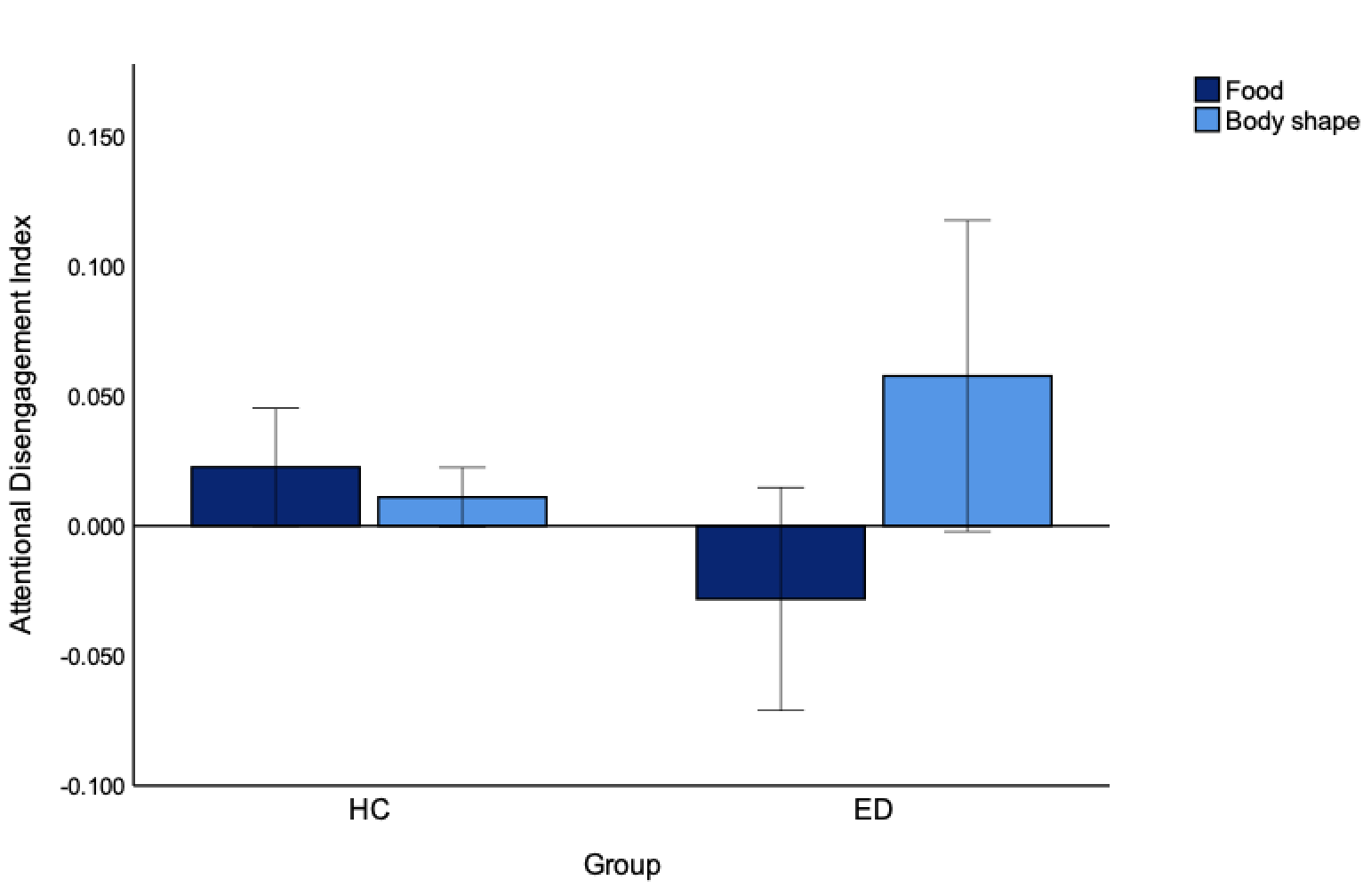


**Figure S5**. Clustered bar graph showing mean AD indexes at 0.2s as a function of cue type and group following mood induction. Error bars denote 95% CI.


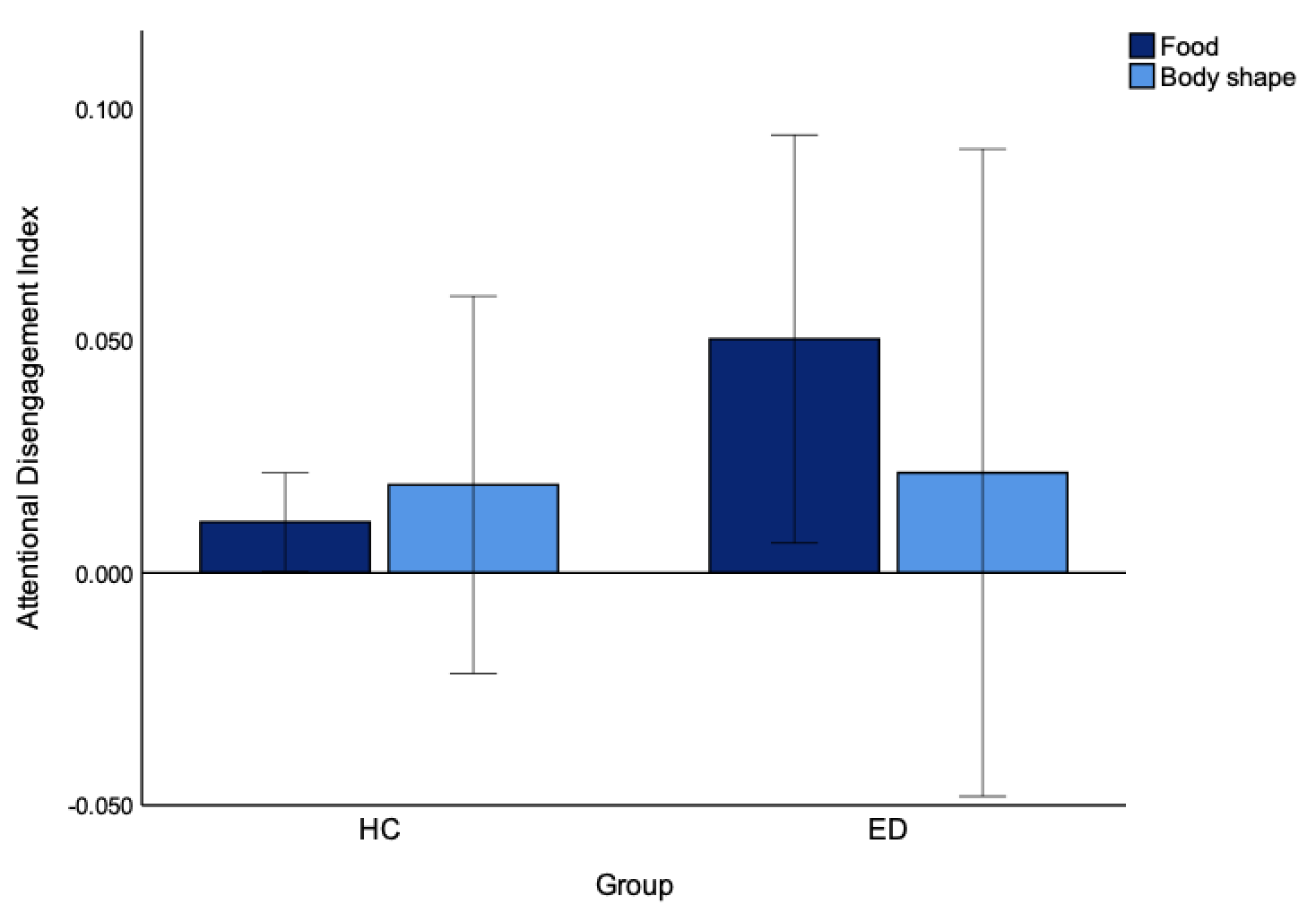


**Figure S6**. Clustered bar graph showing mean AD indexes at 2s as a function of cue type and group following mood induction. Error bars denote 95% CI.

**Table S17.** Output mixed-design ANOVA for AE as a function of cue type and group at presentation times 0.2s and 2s following mood induction adjusting for age, IQ and BMI

|  |  | AE 0.2s | | | | AE 2s | | | |
| --- | --- | --- | --- | --- | --- | --- | --- | --- | --- |
|  | | *df* | *F* | *p* | $\eta_{p}^{2}$ | *df* | *F* | *p* | $\eta_{p}^{2}$ |
| Between-subject effects   age  IQ  BMI  Group  Error (group) | | 1 1 1 1 108 | 3.461 .443  .127 .231 | .066 .507 .723 .632 | .031 .004 .001 .002 | 1  1  1 1 108 | .208 1.210 .001 .063 | .649 .274 .980 .802 | .002 .011 .000 .001 |
| Within-subject effects  Cue type  Cue type x age  Cue type x IQ  Cue type x BMI  Cue type x group  Error (cue type) | | 1  1  1 1 1 108 | 4.745 5.670 .000 .187 2.950 | .032 .019 .988 .667 .089 | .042 .050 .000 .002 .027 | 1  1  1 1 1 108 | 1.759 2.794 .185 .012 5.488 | .187 .098 .668 .914 .021 | .016 .025 .002 .000 .048 |

**Table S18.** Output mixed-design ANOVA for AD as a function of cue type and group at presentation times 0.2s and 2s following mood induction adjusting for age, IQ and BMI

|  |  | AD 0.2s | | | | AD 2s | | | |
| --- | --- | --- | --- | --- | --- | --- | --- | --- | --- |
|  | | *df* | *F* | *p* | $\eta_{p}^{2}$ | *df* | *F* | *p* | $\eta_{p}^{2}$ |
| Between-subject effects   age  IQ  BMI  Group  Error (group) | | 1 1  1  1 108 | 3.167 .318 .322 .000 | .078 .574 .572 .985 | .028 .003 .003 .000 | 1 1 1 1 108 | .199 .902 .018 .331 | .657 .344 .893 .567 | .002 .008 .000 .003 |
| Within-subject effects  Cue type  Cue type x age  Cue type x IQ   Cue type x BMI  Cue type x group  Error (cue type) | | 1 1 1  1  1 108 | 1.456  2.232  .017 .061 2.275 | .230 .138 .897 .805 .134 | .013 .020 .000 .001 .021 | 1 1 1 1 1 108 | .356 .347 .067 .072 1.341 | .552 .557 .796 .789 .249 | .003 .003 .001 .001 .012 |

**Table S19.** Correlations table in ED sample.

|  | EDE-Q | | | | PFS | | | DASS-21 | | |
| --- | --- | --- | --- | --- | --- | --- | --- | --- | --- | --- |
|  | restraint | eating concern | shape concern | global | available | present | tasted | anxiety | depression | stress |
| AE 0.2s  Food | -.003 .981 | .131 .275 | .019 .873 | .037 .760 | .132 .271 | .041 .734 | -.066  .584 | -.069 .564 | .082 .492 | -.039  .745 |
| AE 0.2s  Body Shape | .210 .078 | .067 .579 | **.246* .038** | .198 .098 | **.279* .019** | .173 .149 | .196 .101 | **.031*.010** | **.367** .002** | **.233* .049** |
| AE 2s Food | -.058 .629 | .035  .771 | -.151  .210 | -.062  .608 | .066  .586 | -.101  .400 | -.046  .704 | -.182  .125 | -.061  .612 | **-.235***  **.047** |
| AE 2s Body Shape | .041 .733 | -.095 .432 | -.082 .497 | -.052  .665 | **-.290* .014** | -.054 .657 | -.036 .763 | .106 .376 | .020 .868 | .159 .183 |
| AD 0.2s  Food | -.114 .343 | .214 .073 | .011  .930 | .033 .782 | .107 .375 | .040 .742 | .044 .716 | **-.241* .042** | -.123 .302 | -.119 .321 |
| AD 0.2s  Body Shape | .183 .126 | -.024 .844 | .083 .493 | .096 .427 | .116 .335 | .026 .833 | .105 .384 | **.272*.021** | **.314** .007** | .158 .185 |
| AD 2s Food | -.028 .817 | **-2.41* .043** | **-.235* .049** | -.168 .163 | -.070 .560 | -.127  .291 | -.056 .641 | -.066 .581 | .071 .554 | -.179 .132 |
| AD 2s Body Shape | .010 .934 | .002 .989 | -.065 .591 | -.026 .827 | **-.341** .004** | -.102 .395 | -.073 .547 | .062 .607 | -.052 .663 | .118 .323 |
| EDE-Q restraint | - | - | - | - | .102 .396 | .127 .290 | -.015 .900 | **.566** <.001** | **.505** <.001** | **.512** <.001** |
| EDE-Q eating concern | - | - | - | - | **.439** <.001** | **.373** .001** | .156 .194 | **.420** <.001** | **.479** <.001** | **.458** <.001** |
| EDE-Q shape concern | - | - | - | - | **.411** <.001** | **.429** <.001** | .207 .084 | **.445** <.001** | **.439** <.001** | **.452** <.001** |
| EDE-Q global | - | - | - | - | **.323** .006** | **.334** .004** | .129 .284 | **.506** <.001** | **.524** <.001** | **.503** <.001** |
| PFS available | - | - | - | - | - | - | - | .130 .281 | .209 .080 | .192 .109 |
| PFS present | - | - | - | - | - | - | - | .052 .669 | .148 .219 | .228 .055 |
| PFS tasted | - | - | - | - | - | - | - | .164 .173 | .217 .069 | .188 .115 |

*Note*. Numbers in bold denote significant values.

**Figure S7.** Scatterplot for correlation between AE Body Shape 0.2s and EDE-Q Shape Concern (*r* = .246, *p* = .038).

**Figure S8.** Scatterplot for correlation between AE Body Shape 0.2s and DASS-21 Anxiety (*r* = .301, *p* = .010).

**Figure S9.** Scatterplot for correlation between AE Body Shape 0.2s and DASS-21 Depression (*r* = .367, *p* = .002).

**Figure S10.** Scatterplot for correlation between AE Body Shape 0.2s and DASS-21 Stress (*r* = .233, *p* = .049).

**Figure S11.** Scatterplot for correlation between AE Body Shape 0.2s and POF Available (*r* = .279, *p* = .019).

**Figure S12.** Scatterplot for correlation between AE Food 2s and DASS-21 Stress (*r* = -.235, *p* = .047).

**Figure S13.** Scatterplot for correlation between AE Body Shape 2s and POF Available (*r* = -.290, *p* = .014).

**Figure S14.** Scatterplot for correlation between AD Food 0.2s and DASS-21 Anxiety (*r* = .241, *p* = .042).

**Figure S15.** Scatterplot for correlation between AD Body Shape 0.2s and DASS-21 Anxiety (*r* = .272, *p* = .021).

**Figure S16.** Scatterplot for correlation between AD Body Shape 0.2s and DASS-21 Depression (*r* = .314, *p* = .007).

**Figure S17.** Scatterplot for correlation between AD Food 2s and EDE-Q Eating Concern (*r* = .241, *p* = .043).

**Figure S18.** Scatterplot for correlation between AD Food 2s and EDE-Q Shape Concern (*r* = .235, *p* = .049).

**Figure S19.** Scatterplot for correlation between AD Body Shape 2s and POF Available (*r* = .341, *p* = .004).
